# Severe COVID-19 Pneumonia Complicated by Pneumothorax, Pneumomediastinum, and Pneumoperitoneum

**DOI:** 10.4269/ajtmh.21-0092

**Published:** 2021-04-05

**Authors:** Ashraf O. E. Ahmed, Mouhand F. H. Mohamed, Khalid Ahmed

**Affiliations:** 1 Department of Internal Medicine, Hamad Medical Corporation, Doha, Qatar;; 2 Department of Acute Care Surgery, Hamad Medical Corporation, Doha, Qatar

A 50-year-old man presented with a 4-day history of fever and diarrhea. He experienced shortness of breath on the day of presentation. Physical examination indicated that the patient was in severe respiratory distress. His respiratory rate was 45 breaths/min, and his oxygen saturation was 93% with 15 L of oxygen/minute via a nonrebreathing mask. A chest examination showed diffuse crepitations. Nasopharyngeal swabbing for severe acute respiratory syndrome coronavirus 2 (SARS-CoV-2) yielded positive results. The C-reactive protein and ferritin levels were elevated at 170.9 mg/L (0–5 mg/L) and 5,156.0 μg/L, respectively. The chest X-ray (CXR) examination revealed bilateral lower zone infiltrates.

**Figure 1.**
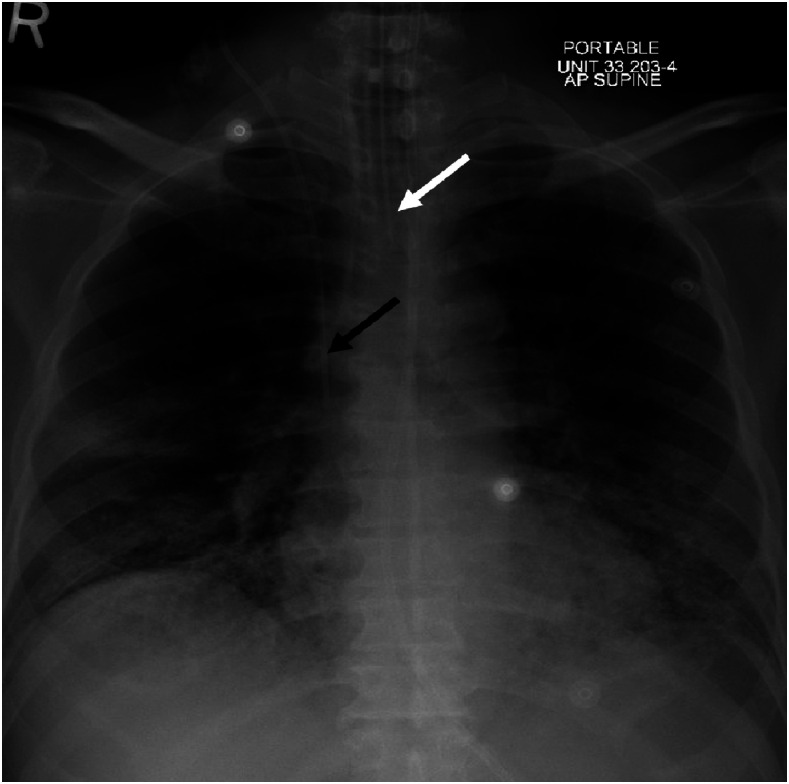
Chest X-ray examination indicating the endotracheal tube (white arrow) and central line insertion (black arrow) with no complications. Repeat demonstration of bilateral infiltrates.

Soon after admission, acute kidney injury developed and hemodialysis was started. Three days later, shortness of breath and desaturation developed; therefore, he was intubated and had a central line inserted (right jugular). The postprocedural CXR examination results were unremarkable (Figure 1).

**Figure 2.**
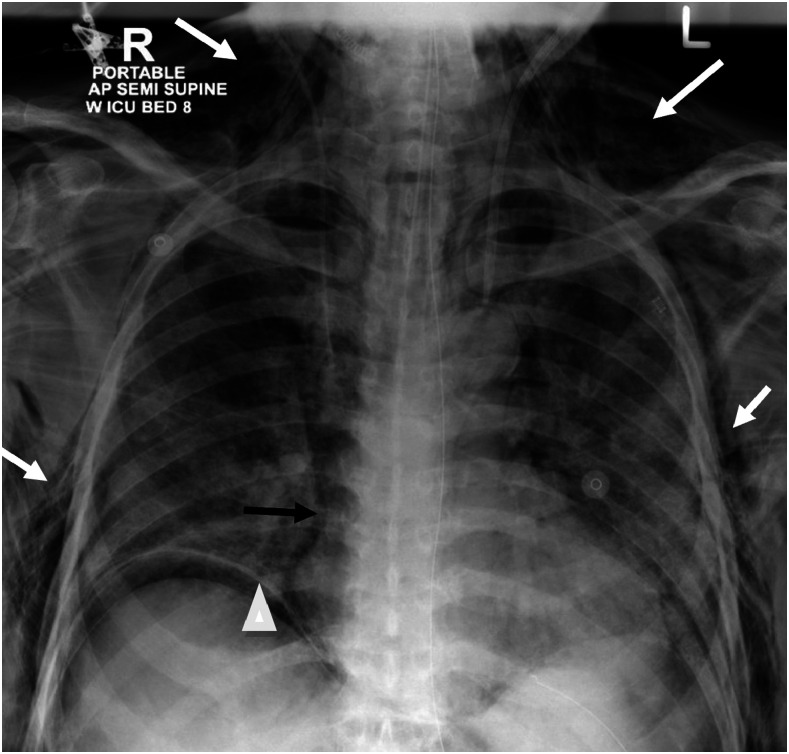
Chest X-ray examination showing surgical emphysema (white arrows), pneumomediastinum (black arrows), and pneumoperitoneum indicated by air under the diaphragm (arrowhead).

**Figure 3.**
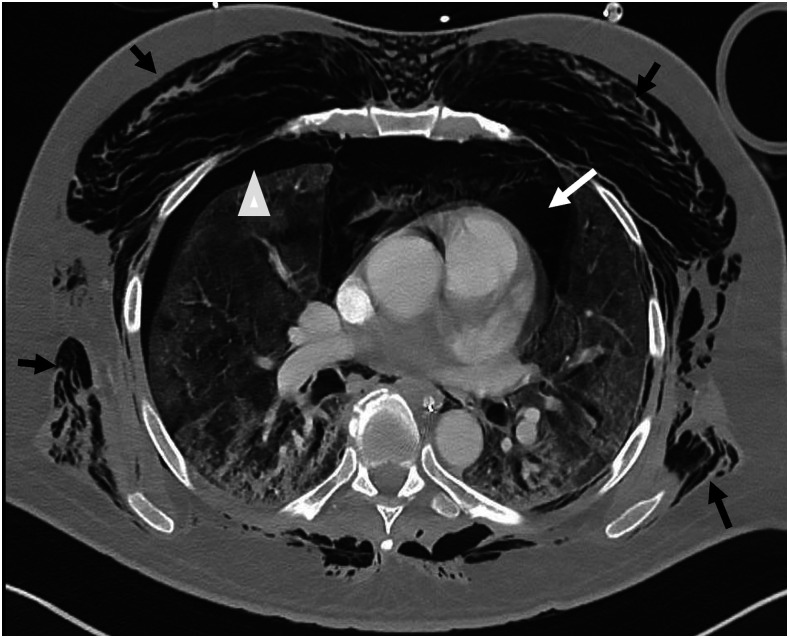
Computed tomography (CT) examination of the chest showing extensive surgical emphysema (black arrows), pneumomediastinum (white arrows), and bilateral pneumothorax (arrowhead).

**Figure 4.**
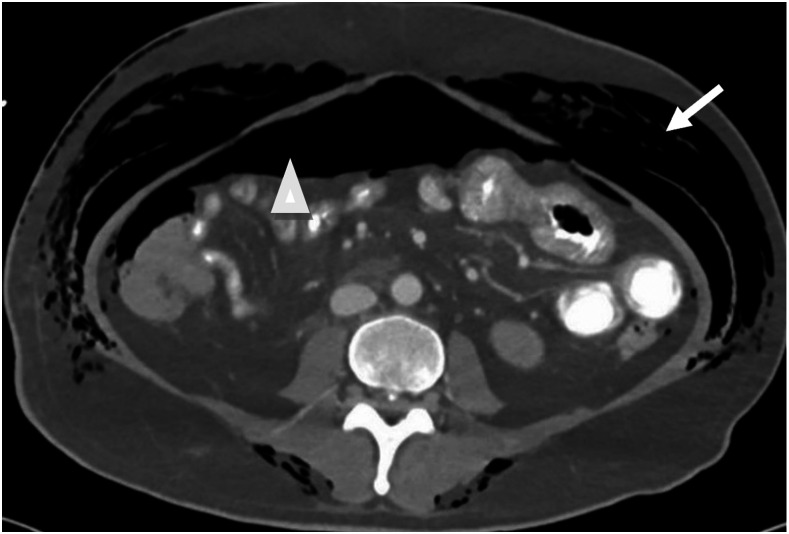
Computed tomography (CT) scan of the abdomen showing extensive surgical emphysema (arrow) and pneumoperitoneum (arrowhead). No free fluid was noted. No signs of peritoneal or mesenteric inflammation were observed.

Three weeks later, on mechanical ventilation, extensive subcutaneous emphysema involving the four limbs, neck, and trunk developed. An additional CXR examination and abdominal computed tomography (CT) examination showed extensive pneumomediastinum with extensive bilateral pneumothorax and extensive pneumoperitoneum (Figures 2–4). A right chest tube was inserted and surgical consultation was sought for pneumoperitoneum. Conservative management was recommended because no contrast extravasation and no signs of peritoneal or mesenteric inflammation were present. After conservative management, progressive improvement was observed. The patient was extubated 5 weeks later. His renal function improved and hemodialysis was stopped.

Coronavirus disease 2019 (COVID-19) has been reported to cause pneumothorax,^1^ which can worsen rapidly under the circumstances of mechanical ventilation because it can act as a shearing force that intensifies air leak into the mediastinum and even to the peritoneum.^2–4^
